# Supplementary material for: Real-time imaging of cellular forces using optical interference
Source: Nat Commun. 2021 Jun 11;12:3552. doi: 10.1038/s41467-021-23734-4 (PMC8196160; doi:10.1038/s41467-021-23734-4)
Supplement: Supplementary file 1 — Supplementary Information [file 41467_2021_23734_MOESM1_ESM.docx]

**Supplementary Information**

**Note 1: Example of Challenges with Single Wavelength Readout**

Interrogating a Fabry-Perot resonator (FPR) with just a single wavelength of illumination suffers from several limitations that make it challenging to determine the FPR thickness. As discussed in the main text, the intensity at each pixel in an image of the reflected light from a low finesse FPR cavity can be approximated by the following cosine equation:

$I\left( \phi\right) \sim B+A\cos2\phi$ , (1)

where$\phi=\frac{2\pi n d}{\boldsymbol{\lambda}}$ . (2)

Using this equation to map the pixel intensity $I\left( \phi\right)$to the resonator thickness *d* presents three main challenges. Firstly, the background term *B* may vary across the field of view or across the cavity because of illumination inhomogeneity. Secondly, the amplitude term *A* varies between different fringes due to resolution constraints, where fringes confined to smaller regions become averaged across fewer pixels. Lastly and most fundamentally, the cosine function is symmetric with respect to an increase/decrease in cavity thickness (i.e. to whether a cell pulls up or pushes down). It is therefore not clear whether a transition from bright to dark in a reflection image corresponds to an increase or decrease in local cavity thickness. All three issues are illustrated in Supplementary Figure 1.


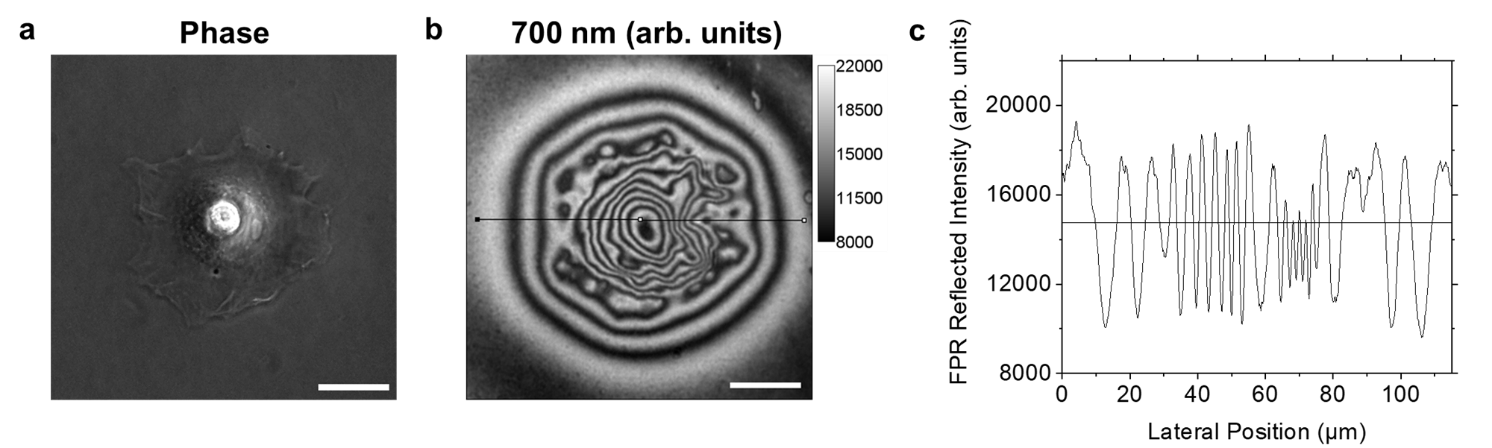


**Supplementary Figure 1:** **a,** Phase-contrast image and **b,** monochromatic reflection image, recorded at 700 nm wavelength of illumination, for a differentiated podocyte adhered to an elastic cavity. **c,** Reflected intensity along the black line in (**b**) showing a reduction in fringe amplitude between 60 and 80 µm lateral position where fringe spacing is closest. A slight variation in background reflected intensity across the image is also visible from the profile plot. Scale bars, 25 μm. Images are representative of a cell from three independent experiments.

**Note 2: Two Wavelength Algorithm Simulation**

While the cosine function (Eq. 1) provides a useful approximation of the reflected light for a low finesse cavity, the reflected light of a FPR microcavity is generally more accurately described by the following equation^1^:

$I(\phi)=I_{inc}\left( 1-\frac{\left( 1-R_{1} \right)\left( 1-R_{2} \right)}{\left( 1-\sqrt{R_{1}R_{2}} \right)^{2}+4\sqrt{R_{1}R_{2}}\sin^{2}(\phi)} \right)$ , (3)

where$\phi=\frac{2\pi n d}{\boldsymbol{\lambda}}$ .

In the following we consider the full equation to investigate the impact of the mirror reflectance and understand the difference from the cosine approximation. The lower the reflectance of the two cavity mirrors, *R*_1_ and *R*_2_, the better the approximation of Eq. 3 by the cosine. Various factors can be explored by first simulating the algorithm (using a software package such as Mathematica), and observing the effect that different parameters have on the interference (Supplementary Figure 2). Higher reflectance not only increases the apparent amplitude of the fringes, but also leads to a sharpening of the minima positions. The typical mirror reflectance for microcavity chips used here is between 10% and 20%.


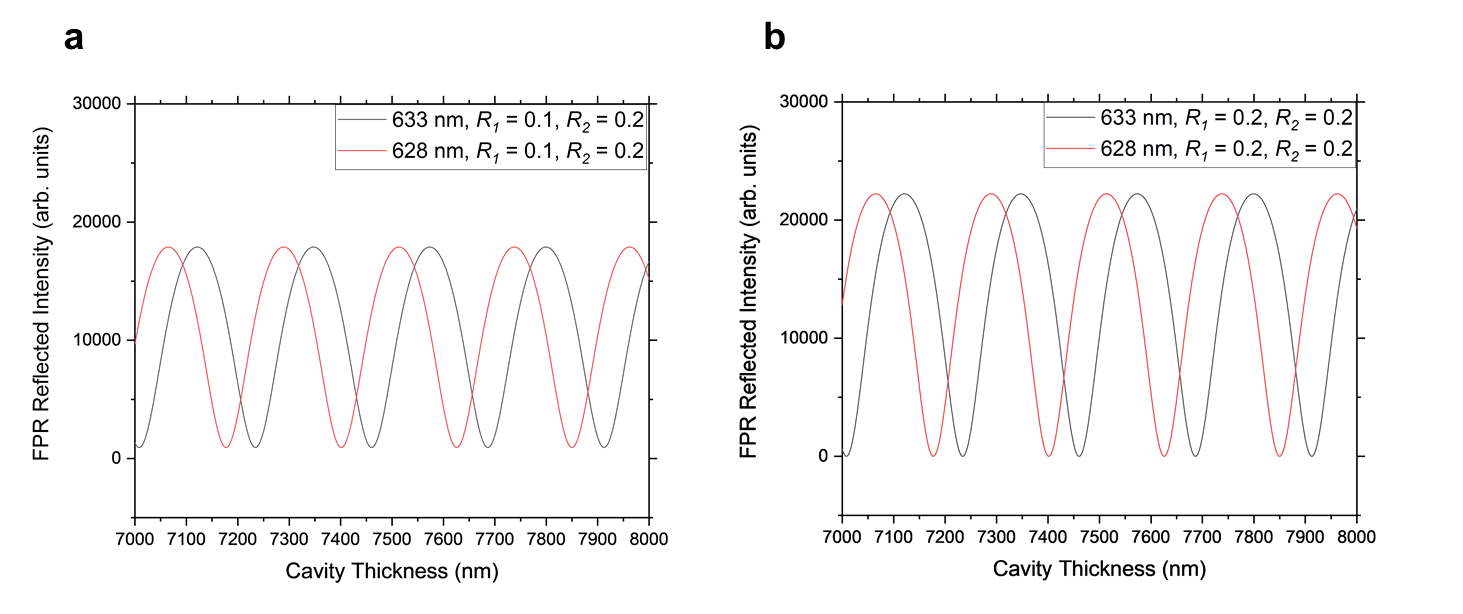


**Supplementary Figure 2: a,** FPR reflected intensity for 633 nm and 628 nm illumination with low mirror reflectance (*R*_1_ = 0.1, *R*_2_ = 0.2). **b,** FPR reflected intensity for 633 nm and 628 nm illumination with slightly higher mirror reflectance (*R*_1_ = 0.2, *R*_2_ = 0.2).

The sharpened minima lead to a sawtooth shape for the fringes in the difference image. Despite this, the sum image remains 90° out of phase, and the maxima and minima still correspond to the zero-crossing points in the difference image (Supplementary Figure 3) which can be used to establish and subtract the background.


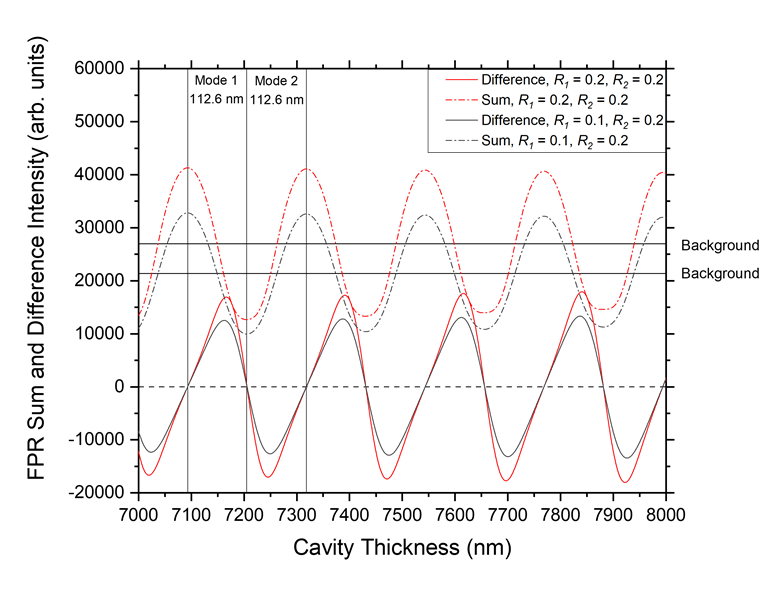


**Supplementary Figure 3:** Sum and difference of reflected intensity at 633 nm and 628 nm, with the regions of two separate modes shown and the background for R_1_ = 0.1 and R_1_ = 0.2 indicated with solid lines.

Upon dividing the sum and the difference curves (Supplementary Figure 4a), the asymmetry of the sawtooth shape results in two unique modes, both with a cotangent shaped profile but each with a slightly different gradient (Supplementary Figure 4b). One mode corresponds to positive values of the difference image (the top half of the sawtooth) while the second mode corresponds to the negative values (the bottom half). Therefore, when translating between cotangent values and displacement, the identity of the mode must be established by referencing the corresponding sign of the values in the difference image.


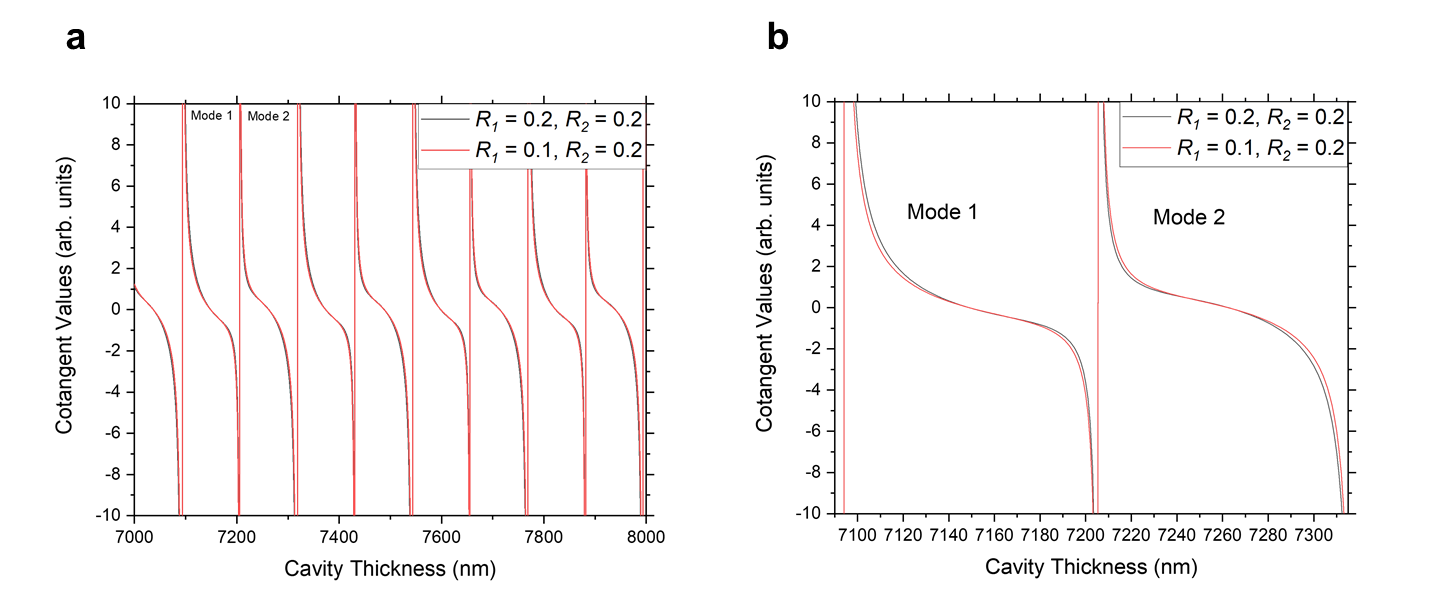


**Supplementary Figure 4: a,** The cotangent shaped profiles generated by the division of the sum and difference functions (after background subtraction). The direction of the cotangent profile reveals the direction of displacement. **b,** Close-up of region in (**a**). The two cotangent modes correspond to positive and negative values of the difference function, respectively. The slope is slightly different between Mode 1 and Mode 2. The two cases, *R_1_* = 0.1, and *R_1_* = 0.2 show very little difference, indicating that the method is robust against variations in reflectance.

While one might intuitively think that it is preferable to maximise the finesse of the cavity to make it most sensitive to cellular force, in practice the 10-20% reflectance of the cavities used in this study is more suitable. As the reflectance of the cavity increases, the asymmetry of the modes becomes progressively stronger (Supplementary Figures 5 and 6). Additionally, the sharper fringes (Supplementary Figure 5a) can lead to artefacts in the minima of the sum image (Supplementary Figure 5b). If the reflectance becomes too high, the small peaks occurring around the minima of the sum image can introduce additional directional artefacts in the cotangent function. Picking wavelengths spaced more closely together (such as 633 nm and 630 nm, instead of 633 nm and 628 nm) can alleviate this issue for high reflectance cavities.


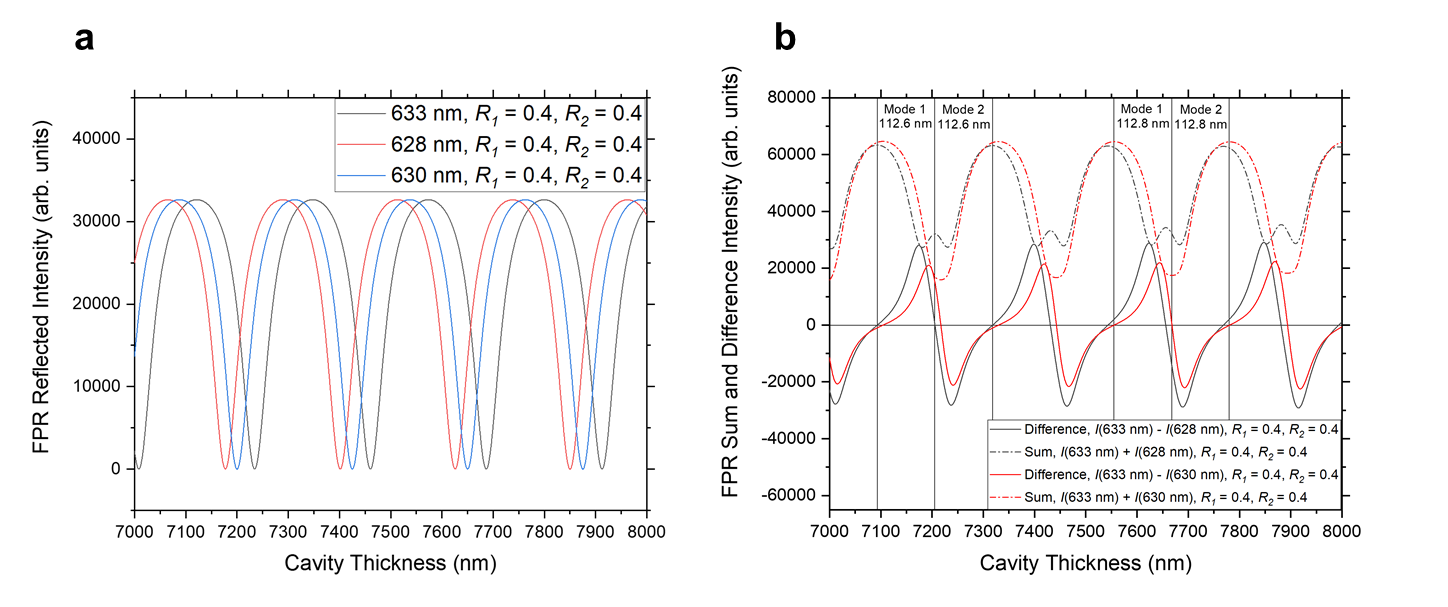


**Supplementary Figure 5: a,** FPR reflected intensity for higher values of *R*_1_ = *R*_2_ = 0.4, for three different wavelengths of illumination: 633 nm, 630 nm, and 628 nm. **b,** The sum and difference functions for the combination of 628 nm with 633 nm, and for the combination of 630 nm with 633 nm. The asymmetric shape becomes more pronounced for the higher values of reflectance shown here (compare to Figure 3). Additionally, a distortion is visible around the minima of the sum function for the combination of 628 nm with 633 nm.

For moderate variations in reflectance (e.g. for a change from 10 to 20%, already much larger than the maximum variation we encounter for nominally identical cavities made in different batches), the shape of the cotangent function does not change in a significant manner, with slopes for the different reflectance values differing by ≈1 nm (Supplementary Figure 4b). However, for *R*_1_ = *R*_2_ = 40 % the slopes show a clear difference from those at a lower reflectance (Supplementary Figure 6). Therefore, if elastic cavities with very different mirror reflectance are used, it is necessary to recalibrate the cotangent curves.


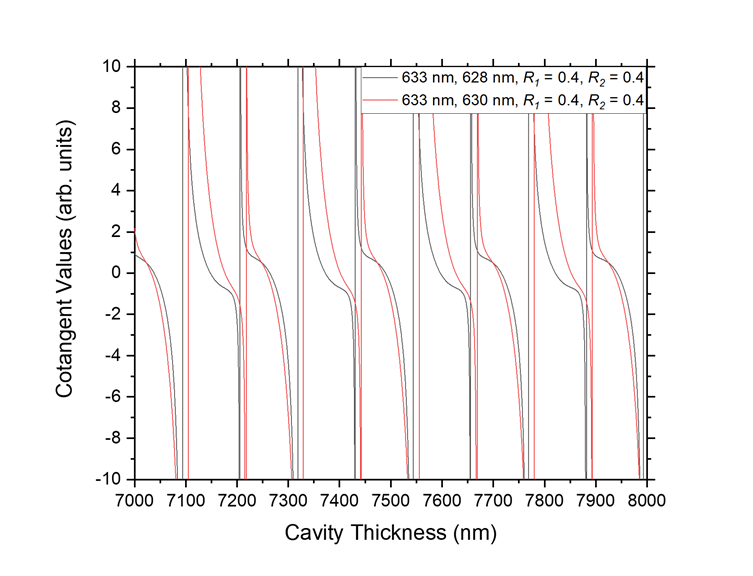


**Supplementary Figure 6:** The cotangent shaped profiles generated by the division of the sum and difference functions for higher reflectance FPRs. The sharper resonances lead to a greater asymmetry between the two modes produced by positive and negative values of the difference function.

Another important feature that affects the shape of the profiles generated by the division of the sum and difference functions is the total thickness of the microcavity, which is 7-8 μm for the cavities used in this study. In addition to the fast phase term *k_fast,_* there is also the slowly varying envelope term *k_slow_* that leads to a ‘beating’ signal when looking at the sum and difference equations over a wide range of cavity thicknesses, i.e. much wider than the few hundred nanometres typically observed due to cell-induced deformations (Supplementary Figure 7).


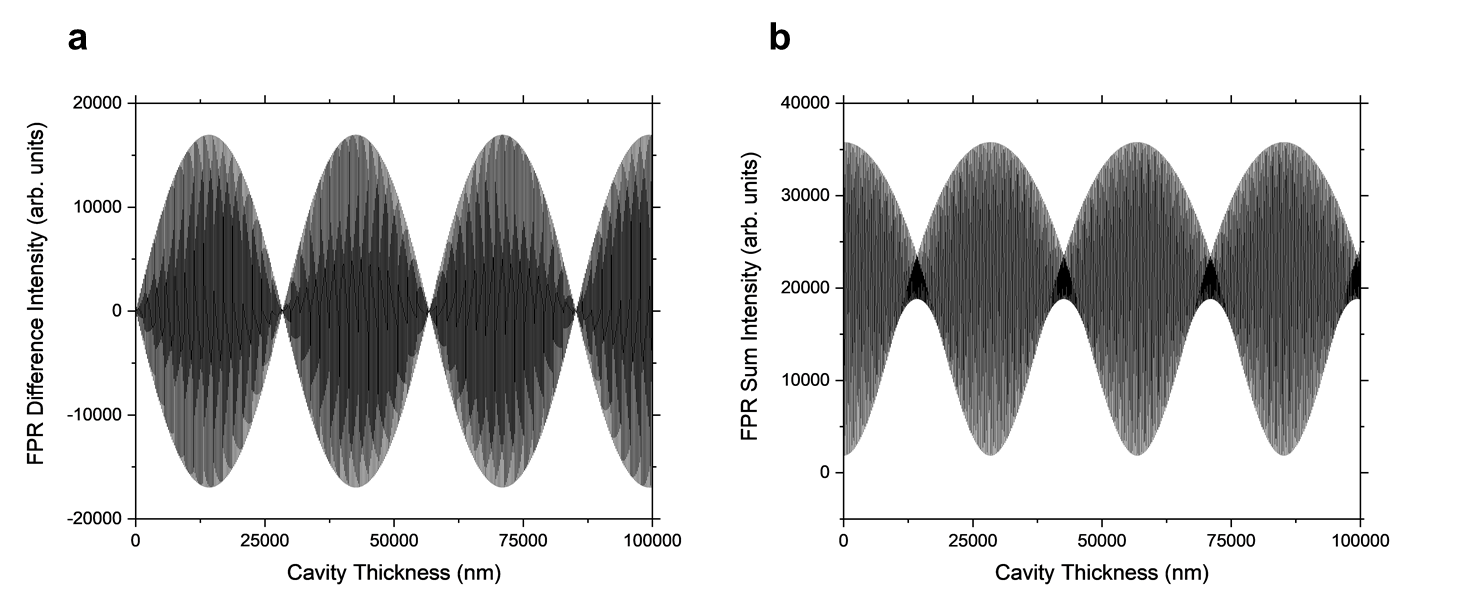


**Supplementary Figure 7: a,** The envelope of the difference function varies slowly over many µm of cavity thickness. This affects the amplitude of the faster phase term. At the zero points, the information in the difference image is lost. **b,** The envelope of the sum function also varies slowly over many micrometres of cavity thickness change. At the zero position points (after background subtraction; at ca. 20,000 in the non-background corrected profile here) the information in the sum image is lost.

However, if the cavity thickness changes by many µm, the envelope term causes a change in the slope of the profiles generated by the division of the sum and difference functions (Supplementary Figure 8a). However, given that cells generally only deform the cavity by a few hundred nanometres, the effect of the envelope term can be neglected (Supplementary Figure 8b).

**
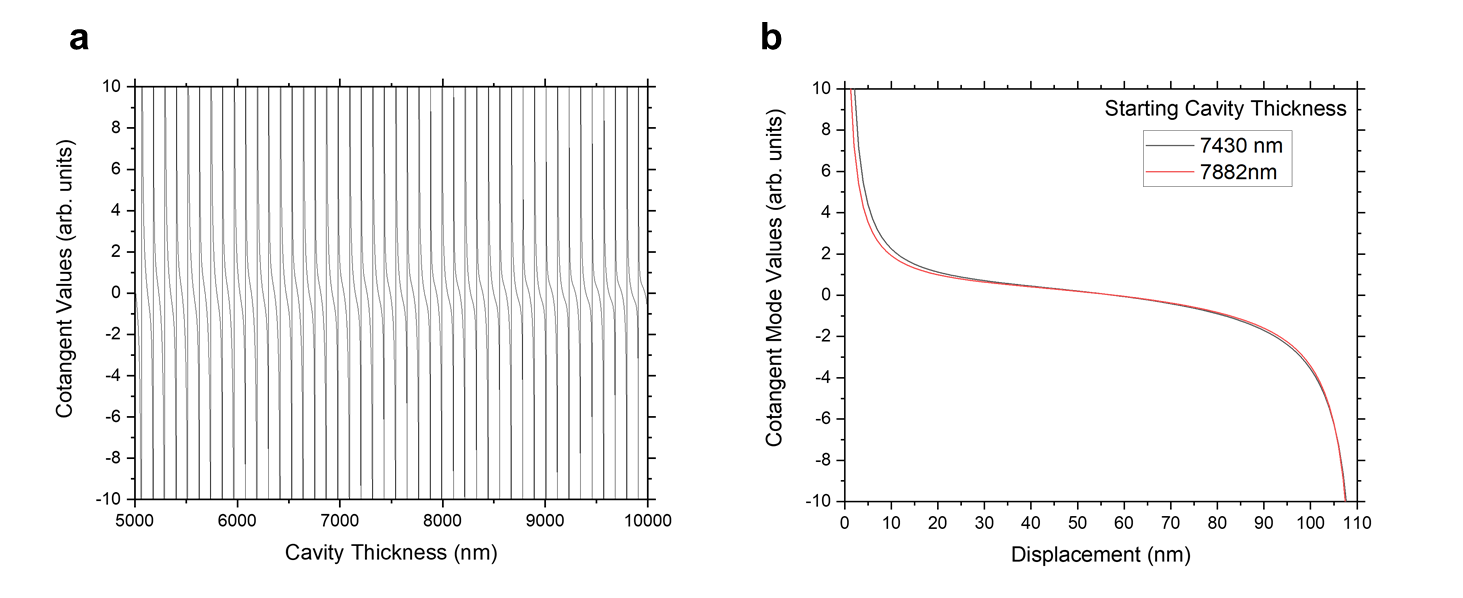
**

**Supplementary Figure 8: a,** The slope of the cotangent shaped profiles generated by the division of the sum and difference functions changes gradually if the cavity thickness changes by many µm. **b,** A comparison between two profiles for cavities differing by ≈450 nm in thickness (the labels indicate the starting thickness, i.e. for 0 nm displacement). This thickness difference corresponds to the maximum cell-induced cavity deformation observed in typical experiments. While there are some small differences, the profiles remain within a few nanometres of each other.

The frequency of the envelope term depends on the spectral separation between the two wavelengths used. The closer together they are, the more slowly varying the resultant envelope. Therefore, the two wavelengths are ideally spaced by one-quarter of the free spectral range (FSR) of the cavity (Supplementary Figure 9a).


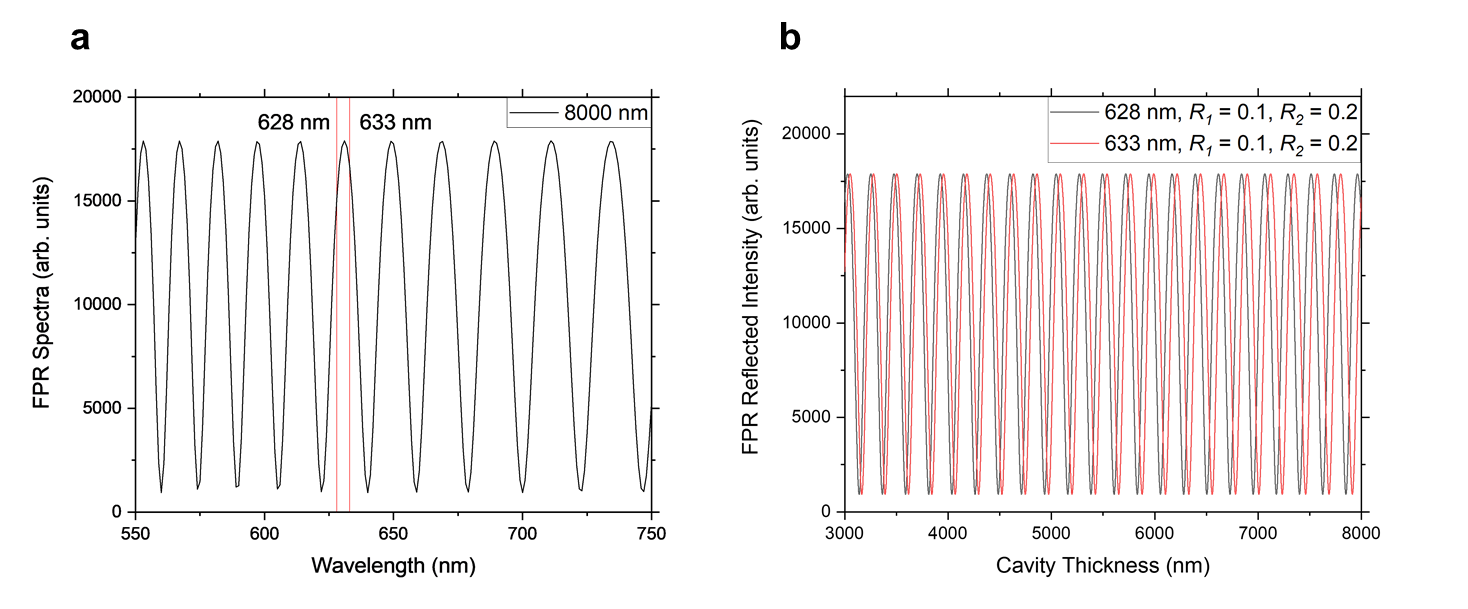


**Supplementary Figure 9: a,** The FPR reflected intensity as a function of wavelength. The two wavelengths used for this study are spaced by ≈1*/*4 of the FSR of the FPR. **b,** FPR reflected intensity versus cavity thickness for two different wavelengths of illumination, showing a gradual shift in phase.

The physical origin of the envelope term and beating in the sum/difference functions is that the two wavelengths move in and out of phase as the cavity thickness changes (Supplementary Figure 9b). It is necessary to take this into account when making an exact choice of wavelength for a measurement. If the wavelengths are fully in phase, then the difference image loses the interference information as the envelope amplitude of the difference function becomes zero, while if they are 180**°** out of phase, the sum image loses all information.

Given the importance of the envelope function for drastic changes in cavity thickness, the calibration of the tangent curves should be performed separately for cavities that are produced with very different elastomer thicknesses. Once the calibration is known, the phase and therefore the displacement can be determined at any future point using the value of the ratio between the sum and difference functions. The information obtained is analogous to scanning through a wide range of wavelengths to determine the phase and resonant peak positions at each point in the field of view. The latter was successfully applied in our prior publications^2,3^, but it requires recording and processing of much more data and is thus intrinsically slower than the approach with just two wavelengths used here.

**Note 3: Comparing the Accuracy of the Wavelength-Alternating Approach to the Wavelength-Scanning Approach**

Supplementary Figure 10a shows the change in reflected intensity for a single pixel within an image stack obtained by scanning the wavelength of illumination over a 201 nm range. The images from this stack taken at 695 nm and 700 nm illumination are presented in Fig. 2 of the main text.

Following the generation of displacement maps with the wavelength-alternating approach developed in the present work (Fig. 2g main text) and with the scanning ERISM approach reported previously^2^ (Supplementary Figure 10b), a difference image of the two maps was created (Fig. 2i in the main text). A histogram of the pixel values of this difference image is shown in Supplementary Figure 10c.


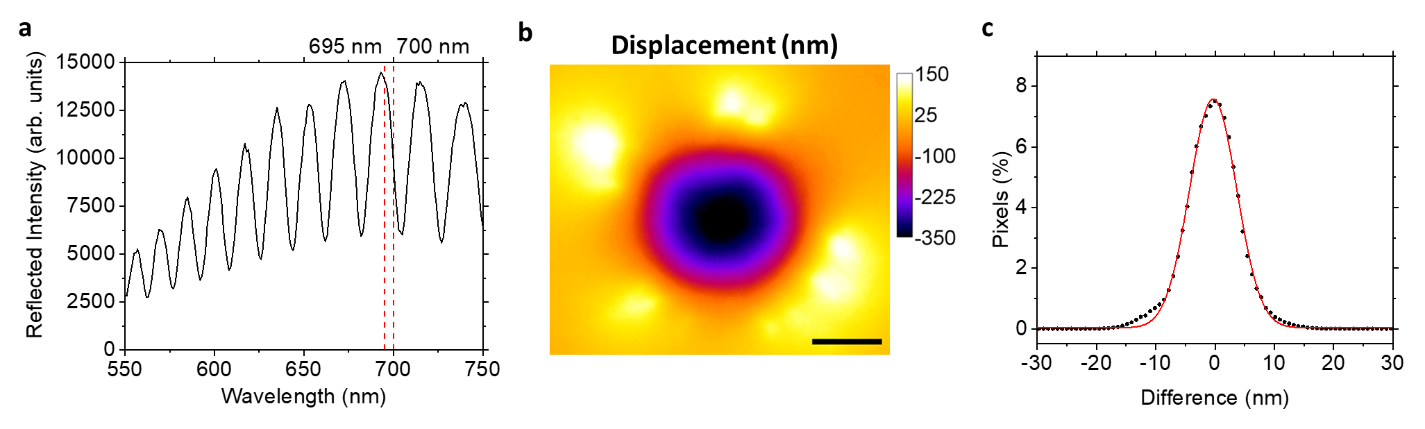


**Supplementary Figure 10: a,** Wavelength scan for a single pixel of an image stack over 201 wavelengths. The red dashed lines indicate the wavelengths used to compute the displacement map from the wavelength-alternating algorithm developed in the present work in Fig. 2 of the main text. **b,** A displacement map created using the unique resonant mode positions from a scan across 201 different wavelengths. **c,** A histogram of the pixel values of the difference map in Fig. 2i of the main text. The full width at half maximum of the histogram is 9.4 nm, corresponding to 2% of the total displacement caused by the cell.

**Note 4: Hertz Model Fit to Podosome Forces**

Supplementary Figure 11 shows the podosomal force found from the stress map time-lapse in Fig. 3e of the main text, plotted against the corresponding depth of podosome protrusion into the microcavity as taken from the displacement map time-lapse in Fig. 3d. Additionally, we used the Hertz model for a hard cylinder indenting into an infinitely thick elastic layer to estimate the podosomal force^4^:

$F=2R\frac{E}{1-ʋ^{2}}d$ .

Here, *R* is the radius of the indenter (taken as the radius of a circle with the same size as the indentation area of the corresponding podosome), $E=0.3\pm0.05$ kPa is the Young’s modulus of the bulk elastomer^2^, and *d* is the depth of indentation (taken from the displacement maps as the maximum indentation under each podosome). The Poisson's ratio of the elastomer $ʋ$ was assumed to be 0.49, a value previously reported for the structurally similar elastomer PDMS^5^.

The forces estimated from the Hertz model are also shown in Supplementary Figure 11, and the FEM and the Hertz model force values are generally similar. Linear fits to both datasets also show good correlation between force and podosome indentation and comparable gradients for both methods (1.07 pN/nm for the FEM data and 0.99 pN/nm for the Hertz model data).

**Supplementary Figure 11:** The podosome force measured from the FEM stress maps (red symbols) and the podosome force estimated via the Hertz model for a cylindrical indenter (grey symbols), both plotted versus the indentation of the respective podosome as taken from the displacement map. The linear fits have R^2^ values of 0.58 and 0.89 for the FEM data and Hertz model, respectively.

**Note 5: Validation of WARP Force Calculation via AFM**

To test the ability of WARP to calculate force from cavity displacement, AFM was used to indent the microcavity chip with a set of known forces. Displacement maps were recorded while the AFM force was held constant (example shown in Supplementary Figure 12a and b). Stress maps were then calculated from the displacement maps via the same FEM used to analyse the podosome data (Supplementary Figure 12c). The known AFM force was then compared to the force calculated from integration of the stress maps (Supplementary Figure 12d). Two different AFM cantilevers with spherical indenters of different sizes (1 µm and 5 µm radius) were used to evaluate the microcavity stiffness over a range of forces. The force predicted by WARP and FEM agree with the AFM force to within <10 %, except for the largest force applied with the 1 µm radius indenter and the smallest force applied with the 5 µm radius indenter. We attribute the larger deviations for these conditions to the fact that a non-linear regime is reached with the small indenter at large force levels^6^, and that the cantilever of the large indenter reaches its sensitivity limit at small forces, respectively.

**
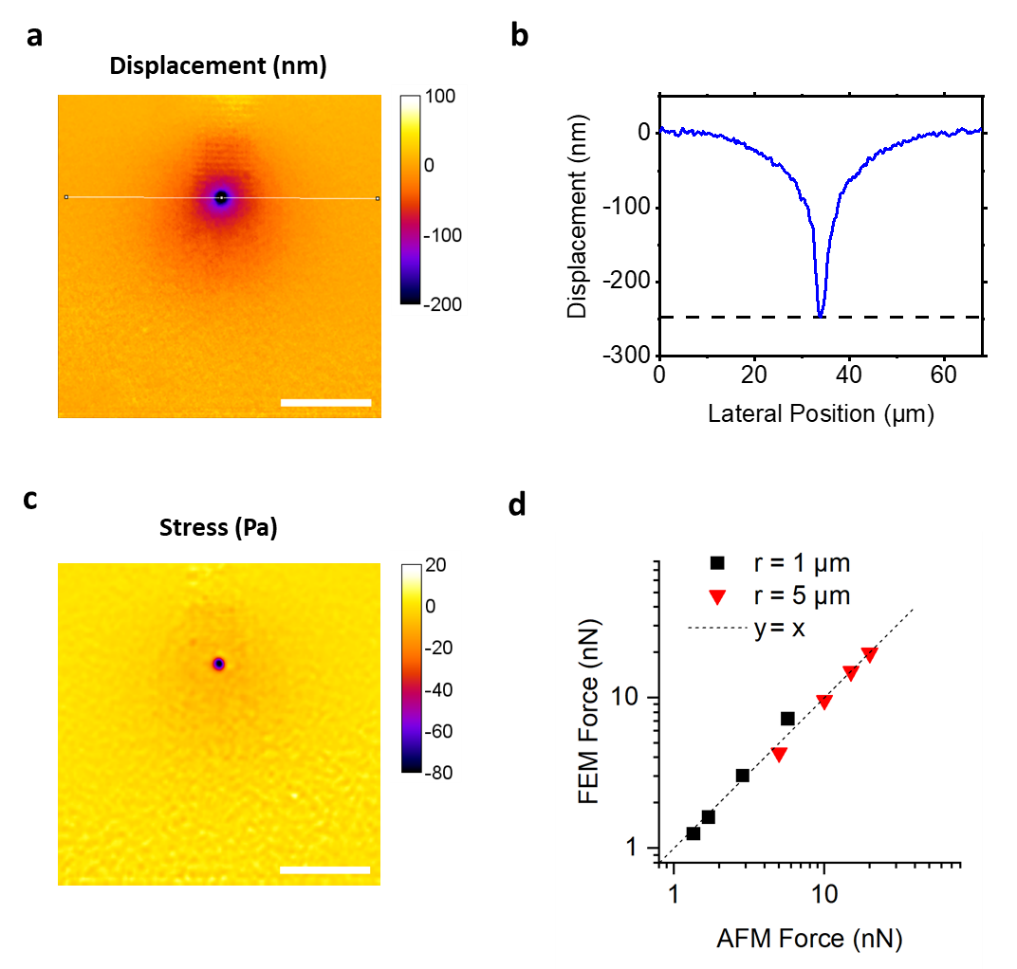
**

**Supplementary Figure 12:** Evaluation of accuracy of force calculation from FEM stress maps. **a,** WARP displacement map for an AFM indentation of 5.7 nN. **b,** Line plot through the centre of the AFM indentation shown in (**a**), with the total indentation reaching a minimum of approximately 250 nm. **c,** Stress map calculated from the displacement map shown in (**a**) using the FEM routine applied for analysis of the podosome data. **d,** Log-log plot of FEM calculated forces against force applied by AFM for two different spherical indenters with r = 1 μm and 5 μm radius, respectively. A line with slope 1 going through the origin is shown as a guide to the eye. Scale bars, 20 μm.

**Note 6: Fourier-Based Frequency Analysis of Rapid Fluctuations in Podosome Force**

Supplementary Figure 13a. shows a Fourier amplitude spectrum of the force-time plot in Fig. 3g (the black line) of the main text. This shows the variation in the force exerted by a macrophage podosome over time. There is clearly a contribution of oscillation frequencies above 0. 5 Hz, though we do not identify a dominant frequency.

As a control, we also analysed the temporal evolution of force in a region of identical size outside the cell interior (Supplementary Figure 13b). As for Fig. 3g in the main text, a 0.5 s moving average filter was applied. Here, no rapid variations in force are observed, and the magnitude of the force is lower than for the area with a podosome. When also applying the 0.2 Hz high-pass filter, no force is visible anymore, in contrast to the situation for the podosome in Fig. 3g of the main text. To confirm this difference further, Supplementary Figure 13c shows the Fourier amplitude spectrum of the black line in Supplementary Figure 13b, indicating clearly that there are no substantial force oscillations. Supplementary Figure 13d. shows the spectra in dB for the regions with (in red) and without (in blue) a podosome for comparison. Supplementary Figure 13e shows a frame from the podosome stress time-lapse series, indicating the areas over which stress was integrated to provide the force in Fig. 3g of the main text and in Supplementary Figure 13b below. A threshold to limit the analysis to negative values of stress was applied to the white region encompassing the podosome. A similar threshold was used to isolate individual podosomes for the force-area plot in Fig. 3h of the main text.


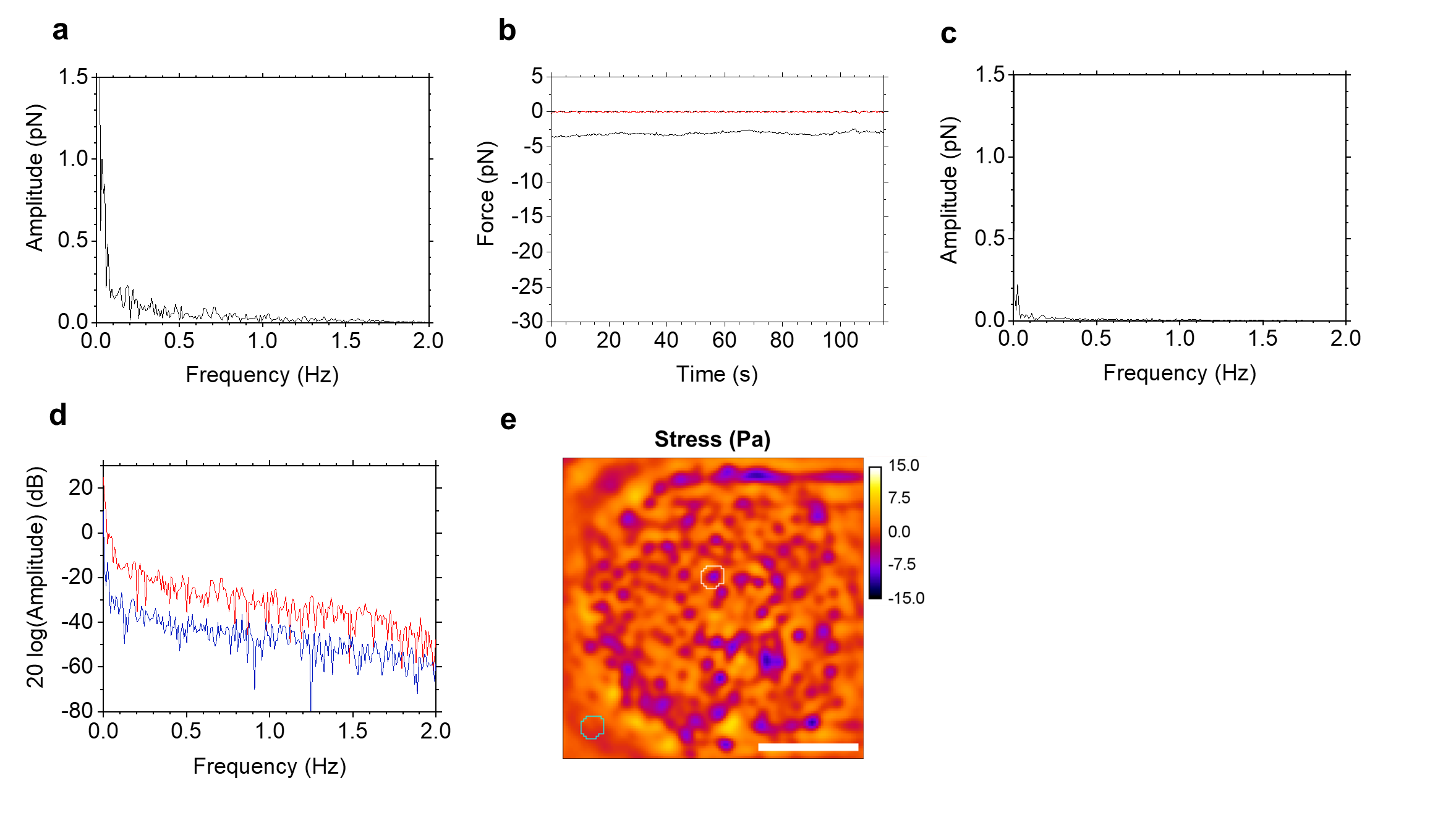


**Supplementary Figure 13: a,** Fourier spectrum of the non-filtered temporal evolution of podosomal force in Fig. 3g of the main text. **b,** Force over time in a region outside the macrophage podosomes in Fig. 3e of the main text. As for the data in the main text, a 0.5 s moving average filter was applied (black). After high pass filtering of the data (cut-off 0.2 Hz) no forces are visible anymore (red). The force is negative indicating it is directed opposite (upwards) compared to the downward pushing force exerted by the podosome in Fig. 3g of the main text. **c,** Fourier spectrum of the non-filtered data in (**b**). **d,** The amplitude in dB for the spectra in (**a**) (red) and (**c**) (blue). **e,** A frame from the macrophage stress time-lapse in Fig. 3e of the main text. The white outline indicates the area over which stress was integrated to obtain the podosomal force. The cyan outline indicates the control area over which stress was integrated to obtain the typical force dynamics in the absence of podosomes, outside the cell interior. Scale bar, 10 μm. Image is representative of three observations from a single experiment.

**Note 7: Statistical Analysis of the Effect of Nifedipine on Cells**

Data from the nifedipine challenge of cardiomyocytes was analysed for *n* = 4 cells (Supplementary Figure 14). All cells showed a significant reduction in contraction force after addition of nifedipine and there was a moderate recovery after washout in most cases. Contraction frequency increased moderately so that typically cells exhibited shallower and faster beats, following the addition of nifedipine. One cell stopped beating entirely upon nifedipine addition but recovered after the washout.


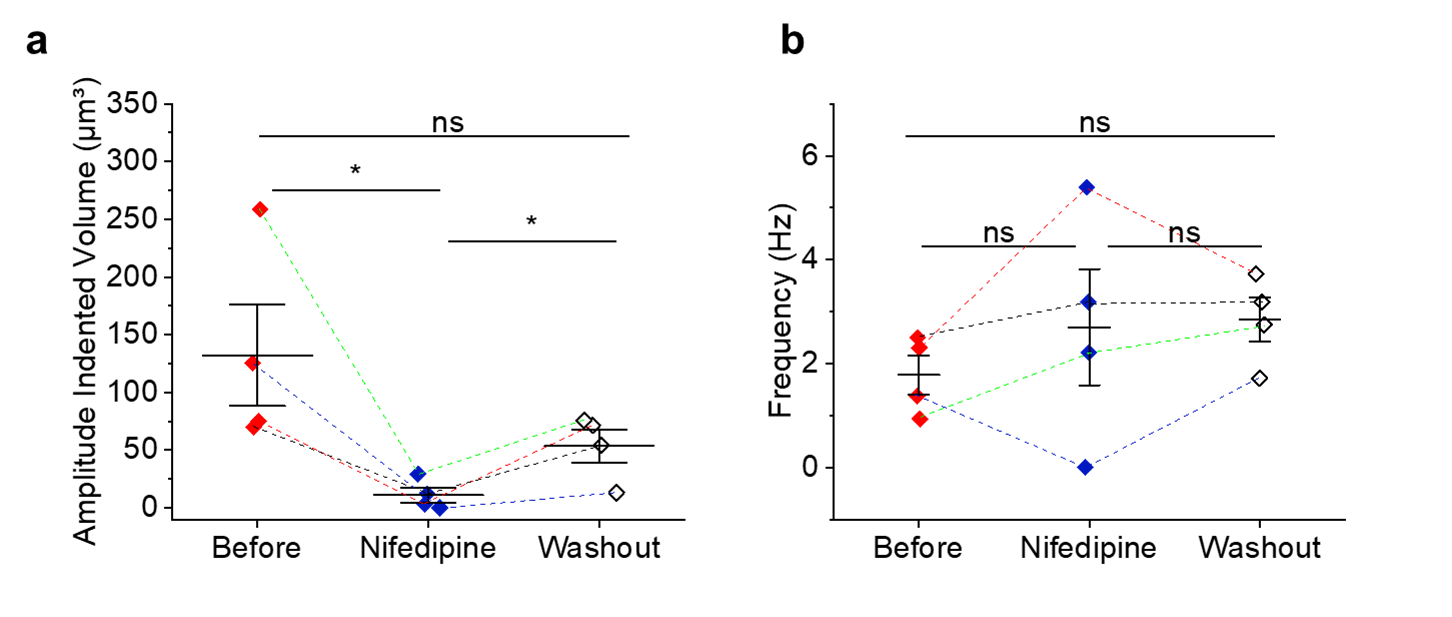


**Supplementary Figure 14: a,** Amplitude of the indented volume for *n* = 4 cells before, during, and after nifedipine challenge. Symbols indicate data, centre line indicates mean, whiskers indicate standard error of the mean (s.e.m.). One-tailed Mann-Whitney *U*-test, *P* < 0.05 (*), *P* > 0.05 (ns). (Before-Nifedipine: *P* = 0.014, Before-Washout: *P* = 0.1, Nifedipine-Washout: *P* = 0.029.) **b,** Scatter plot showing the effect of the nifedipine challenge on the contraction frequency for *n* = 4 cells. Symbols indicate data, centre line indicates mean, whiskers indicate the s.e.m. (One cell stopped beating entirely during the nifedipine challenge, which is indicated as zero frequency in the plot, but recovered during washout.) One-tailed Mann-Whitney *U*-test, *P* > 0.05 (ns), (Before-Nifedipine: *P* = 0.34, Before-Washout: *P* = 0.057, Nifedipine-Washout: *P* = 0.6.)

**Note 8: Cardiomyocyte Force-Frequency Relationship**

The relationship between cardiomyocyte force and contraction frequency is summarised in Supplementary Figure 15, comparing microcavities of different surface stiffness. This further expands on the data discussed as part of Fig. 4 of the main text. For both surface stiffnesses, there is a negative correlation between the amplitude of the indented volume and the contraction frequency; Pearson correlation coefficient -0.50 (14 kPa) and -0.47 (6 kPa). The slope of linear fits to the data is steeper for the stiffer surface; -44.1 μm^3^ Hz^-1^ (14 kPa) and -13 μm^3^ Hz^-1^ (6 kPa).

**Supplementary Figure 15**: Analysis of correlation between the amplitude of the indented volume during cardiomyocyte contraction and the contraction frequency for two different apparent substrate stiffnesses. The lines represent linear fits to the data; R^2^ = 0.25 (14 kPa) and 0.22 (6 kPa).

**Note 9: Long-Term Measurements on a Cluster of Cardiomyocytes**

A cluster of cardiomycoytes consisting of three cells, which was imaged over the course of an hour, is shown in Supplementary Figure 16a-c. The indented volume in the central region of the top cell (Cell 1, c.f. the region contained by the two white lines in Supplementary Figure 16b) was analysed in detail in Fig. 5c-e of the main text. Supplementary Figure 16d-g shows sections of the 1-h dataset in varying degrees of detail; Supplementary Figure 16e is the section shown in the main text; Supplementary Figure 16g shows an even finer timescale, illustrating the background to the force-frequency correlation graph in Fig. 5e of the main text. Cell 1 also shows micro-contractions during the resting phase between contractions as can be seen in Supplementary Video 6, which shows 20 s of the 1 h measurement.


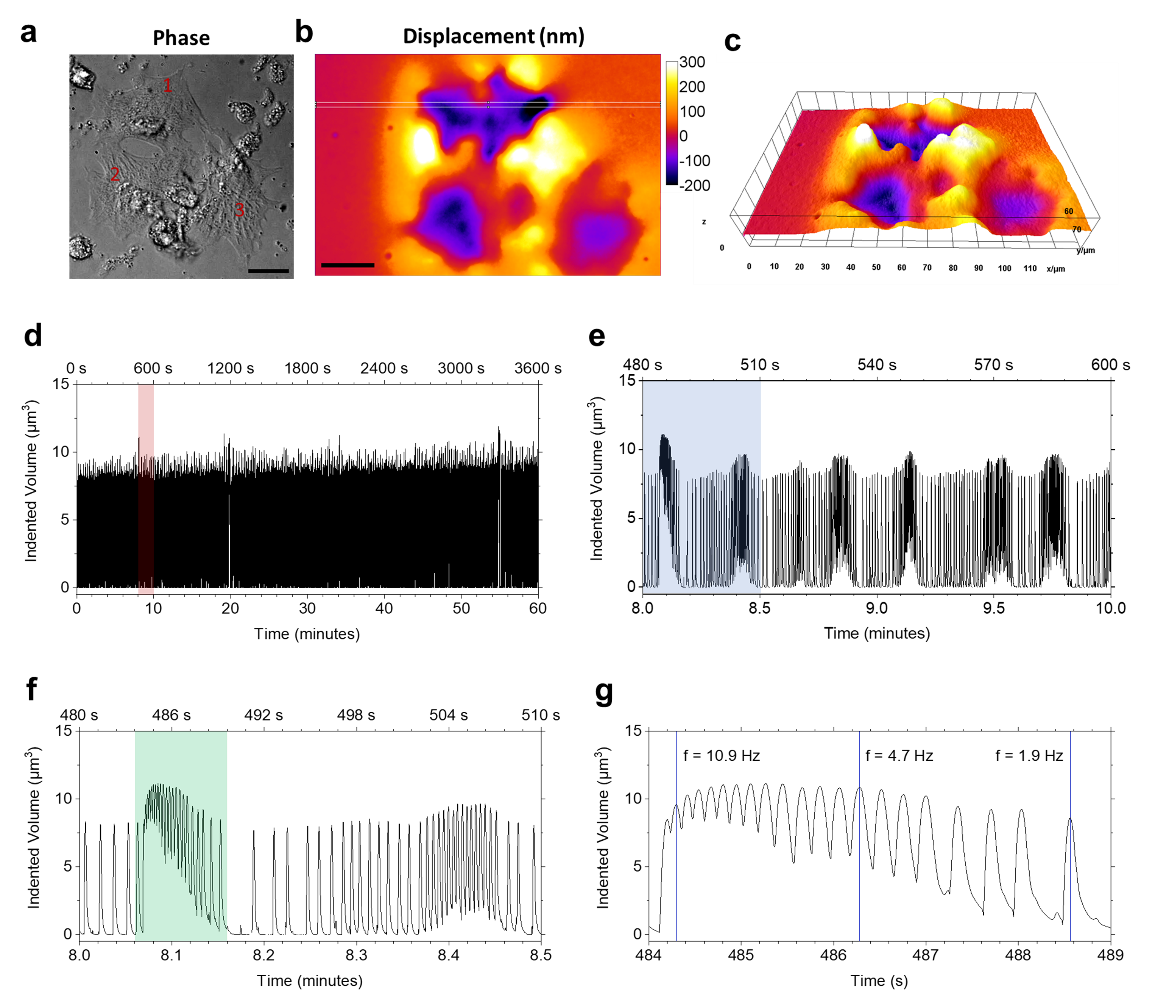


**Supplementary Figure 16: a,** Phase-contrast image of a network of three cardiomyocytes. **b,** The corresponding displacement map showing the contraction of the three cells is in unison during the beating cycle. **c,** 3D rendering of the same displacement map shown in (**b**). **d,** The indented volume for the section under Cell 1 that is indicated by the region within the white lines in (**b**) over the course of one hour. **e,** A magnification of the data in (**d**) over the two-minute period highlighted by the red box in (**d**). **f,** A magnification of the data indicated by the blue box in (**e**) over a 30 s period. **g,** Magnification of the data in (**f**) highlighted by the green box. The numbers next to the blue lines indicate the frequency of corresponding specific contractions. Scale bars, 20 μm. Images are representative of three observations of cell networks from one experiment.

**Note 10: Experimental Setup for WARP**


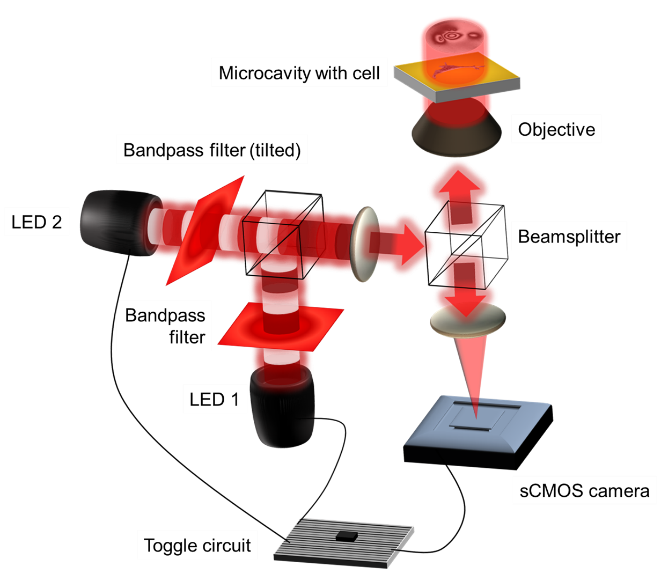


**Supplementary Figure 17:** Schematic of the WARP measurement setup and light path. The two LEDs are alternately triggered to illuminate the microcavity chip with monochromatic light at 633 nm and 628 nm. The resulting reflected interference patterns are captured on the sCMOS camera.

**Note 11: Mechanical Characterization of Microcavity Chips**


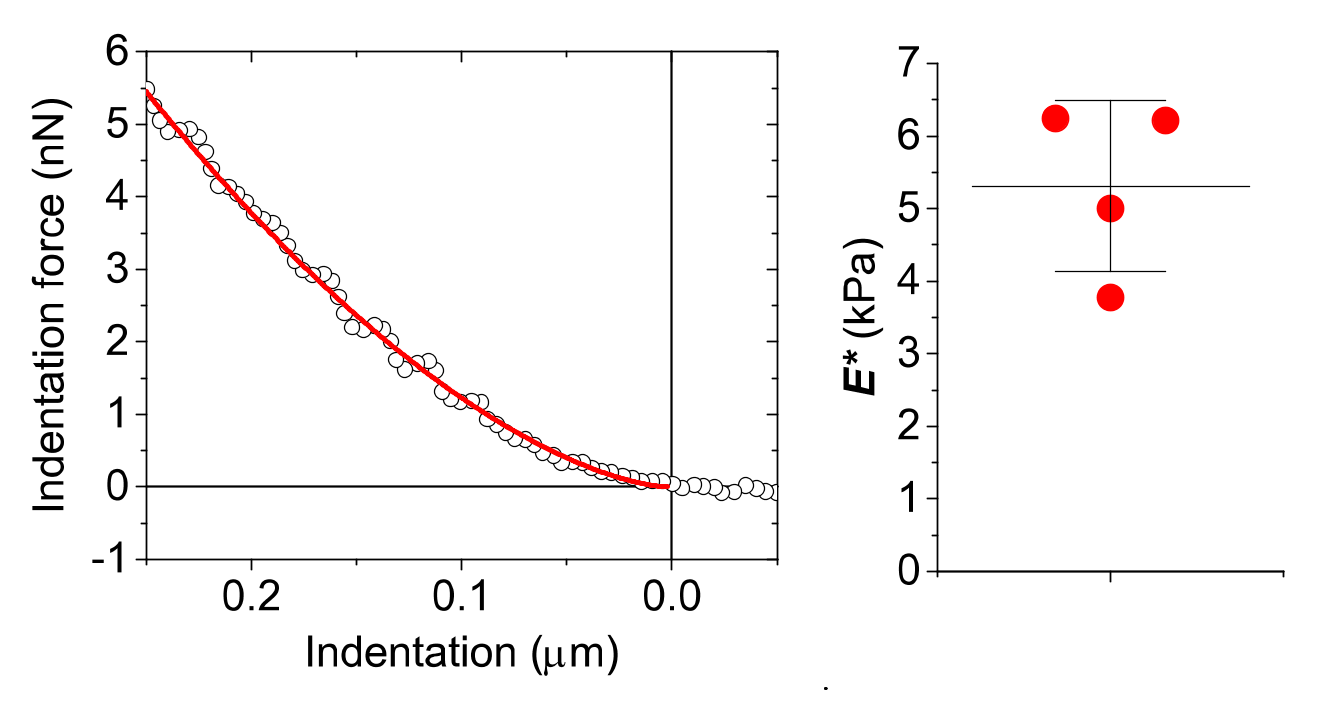


**Supplementary Figure 18:** Mechanical characterization of the elastic microcavity chips used in this work by atomic force microscopy (AFM). **a,** Typical force-distance curve (open symbols) recorded for indentation into the microcavity with an AFM cantilever to which a glass bead with a diameter of 18.2 µm was attached. Data was analysed by fitting with a corrected Hertz model^6^ to obtain the apparent stiffness of the chip (red line). The Poisson's ratio of the elastomer was assumed to be 0.49, a value previously reported for the structurally similar elastomer PDMS^5^. **b,** Measured apparent stiffness of the chip at an indentation force of 1 nN for *n*= 4 AFM measurements taken at different positions across the top mirror surface. Symbols indicate data, centre line indicates mean, whiskers indicate the s.d.

**Supplementary Information References**

1. Ismail, N., Kores, C. C., Geskus, D. & Pollnau, M. Fabry-Pérot resonator: spectral line shapes, generic and related Airy distributions, linewidths, finesses, and performance at low or frequency-dependent reflectivity. *Opt. Express* **24**, 16366–16389 (2016).

2. Kronenberg, N. M. *et al.* Long-term imaging of cellular forces with high precision by elastic resonator interference stress microscopy. *Nat. Cell Biol.* **19**, 864–872 (2017).

3. Liehm, P., Kronenberg, N. M. & Gather, M. C. Analysis of the Precision, Robustness, and Speed of Elastic Resonator Interference Stress Microscopy. *Biophys. J.* **114**, 2180–2193 (2018).

4. Popov, V. L., Heß, M. & Willert, E. Normal Contact Without Adhesion. in *Handbook of Contact Mechanics: Exact Solutions of Axisymmetric Contact Problems* 5–66 (Springer Berlin Heidelberg, 2019).

5. Pritchard, R. H., Lava, P., Debruyne, D. & Terentjev, E. M. Precise determination of the Poisson ratio in soft materials with 2D digital image correlation. *Soft Matter* **9**, 6037–6045 (2013).

6. Dimitriadis, E. K., Horkay, F., Maresca, J., Kachar, B. & Chadwick, R. S. Determination of elastic moduli of thin layers of soft material using the atomic force microscope. *Biophys. J.* **82**, 2798–2810 (2002).
