# Supplementary material for: Real-time imaging of cellular forces using optical interference
Source: Nat Commun. 2021 Jun 11;12:3552. doi: 10.1038/s41467-021-23734-4 (PMC8196160; doi:10.1038/s41467-021-23734-4)
Supplement: Supplementary file 3 — Description of Additional Supplementary Files [file 41467_2021_23734_MOESM3_ESM.docx]

**Description of Additional Supplementary Files**

**Supplementary Video 1:** The components of the WARP microscopy setup. This includes the elastic microcavity chip with a silicone chamber on top containing NIH-3T3 fibroblast cells, which has been placed inside an onstage incubator. The illumination arm consisting of two alternatingly triggered LEDs. Finally, the readout and analysis software interface are shown, displaying the cell displacement maps in real-time in parallel to the phase-contrast images on an adjacent screen. During the measurement, sliders are adjusted to vary both the image contrast and the background subtraction.

**Supplementary Video 2:** Real-time synchronized phase-contrast microscopy (left) and WARP measurement (right) of NIH-3T3 fibroblast cells, each taken at 10.5 fps. Horizontal streaks occur as a result of mode jumps, where the software has not correctly detected a fringe transition in the raw interference data before conversion to displacement. This is corrected for by adjusting the background subtraction. The horizontal streaking effect is more pronounced in real-time data than in datasets that are processed offline as the background can be calculated precisely in the latter. (Colour bar shows displacement in nm.)

**Supplementary Video 3:** Real-time stress measurement of macrophage podosomes. The original dataset was taken at 17 fps, but the time-lapse shown here is sped up approximately 30-fold. While the original WARP maps were taken in realtime, the final conversion to stress was completed offline using FEM. An additional spatial Fourier filter was applied to clearly highlight the nm scale podosome oscillations. (Colour bar shows stress in Pascals.)

**Supplementary Video 4:** WARP measurement of a neonatal murine cardiomyocyte cell recorded at 100 fps. The video has been slowed 10-fold to show the correlation more clearly between the slight contraction in the brightfield video on the lefthand side, and the very distinct variations in displacement in the WARP map. (Colour bar shows displacement in nm.)

**Supplementary Video 5:** WARP measurement of SPOC waves flowing through neonatal murine cardiomyocytes, with false-colour map (left) and a three-dimensional rendering that shows an exaggerated illustration of the displacement (right). Data was acquired at 100 fps and slowed 10-fold to more clearly reveal the presence and form of the contractile waves. Fourier filtering has been applied to remove the strong periodic contractions, leaving the smaller scale SPOC fluctuations occurring during the resting phase in the beating cycle. (Colour bar shows displacement in nm.)

**Supplementary Video 6:** WARP measurement of three cardiomyocyte cells beating in unison, with false-colour map (left) and a three-dimensional rendering that shows an exaggerated illustration of the displacement (right). Data was acquired at 100 fps and has been slowed 10-fold. Micro contractions are present between the main contractile beats, as is most evident in the threedimensional rendering. (Colour bar shows displacement in nm).
